# Object view in spatial system dynamics: a grassland farming example

**DOI:** 10.1080/14498596.2015.1132641

**Published:** 2016-04-25

**Authors:** Christian Neuwirth, Barbara Hofer, Andreas Schaumberger

**Affiliations:** ^a^ Department of Geography, University of Munich, Munich, Germany; ^b^ Doctoral College GIScience, University of Salzburg, Salzburg, Austria; ^c^ Department of Geoinformatics - Z_GIS, University of Salzburg, Salzburg, Austria; ^d^ Department of Geo-Information, AREC Raumberg-Gumpenstein, Irdning, Austria

**Keywords:** Spatial system dynamics, SD and GIS, spatial structure, object geometry and topology, agriculture

## Abstract

Spatial system dynamics (SSD) models are typically implemented by linking stock variables to raster grids while the use of object representations of human artefacts such as buildings or ownership has been limited. This limitation is addressed by this article, which demonstrates the use of object representations in SSD. The objects are parcels of land that are attributed to grassland farms. The model simulates structural change in agriculture, i.e., change in the size of farms. The aim of the model is to reveal relations between structural change, farmland fragmentation and variable farmland quality. Results show that fragmented farms tend to become consolidated by structural change, whereas consolidated initial conditions result in a significant increase of fragmentation. Consolidation is reinforced by a dynamic land market and high transportation costs. The example demonstrates the capabilities of the object-based approach for integrating object geometries (parcel shapes) and relations between objects (distances between parcels) dynamically in SSD.

## 1. Introduction

Process modelling aims at simulating forces and mechanisms driving processes. Process models therefore provide insights into the dynamics of change. Spatial processes interact with space and lead to observable patterns and distributions in space (Thomas & Huggett 1980). The relationship between process and space or structures in space such as transportation networks is often bi-directional. Considering the relationship between process and space in both directions is a challenge in modelling (Birkin 2013). This article presents the application of an object-based spatial system dynamics (SSD) approach that meets this challenge.

As opposed to modelling approaches such as agent-based or cellular automaton modelling, system dynamics (SD) evolved as a non-spatial technique. System dynamics has the strength to consider feedback between model components. The modelling approach has been used for modelling physical and social phenomena (e.g. Gastélum *et al*. 2010; Khan *et al*. 2009). However, as the independent variable in the ordinary differential equations of SD models is always time, space is not implicitly contained in the model formulation.

To overcome the limitation of SD, a new spatial system dynamics approach based on the association of SD stock variables and raster data from geographic information systems (GIS) was presented by Maxwell and Costanza (1997) and applied in numerous studies (e.g. Ahmad & Simonovic 2004; BenDor & Metcalf 2006; Jiao & Xu 2013). This approach foresees a one-to-one raster cell to stock relation. In this way processes modelled as flows between stocks can be visualised in a two-dimensional regular grid. A comprehensive review of techniques can be found in Neuwirth *et al*. 2015.

As opposed to field-based representations, little attention has been paid to the object view of space. This restricts the application of the existing approach in cases where space is better represented by a collection of discrete entities. According to Burrough and McDonnell (1998, p. 32) ‘most human phenomena (houses, land parcels, administrative units, roads, cables, pipelines, agricultural fields in Western agriculture) can be handled best using the entity approach’.

The approach presented in this paper supplements the established method by incorporating irregular spatial arrangements in SD simulations. Furthermore, space itself is modelled dynamically as a function of processes acting upon it. In this way the evolution of man-made structures can be modelled using the well-known SD logic.

As proof of concept, the object-based SSD approach is applied to a model of structural change in grassland agriculture. Structural change in agriculture refers to the change in the number of farms in different farm types classified according to their size, age cohorts or specialisation classes (Zimmermann *et al*. 2006). The changing structure is especially characterised by an increase in average farm size (Goddard *et al*. 1993). In this study particular attention was dedicated to farmland competition and scaling-up of enterprises as one aspect of structural change as well as its spatial implications. The conceptualisation of this agricultural system suggests a model based on an object view considering feedback between process and space:

• the simulation environment is made up of a number of plots (polygon objects) that belong to farms;
• ownership of plots changes in the course of time and is a property of plots that needs to be repeatedly updated;
• ownership patterns affect transportation costs, machinability of farms, economic yield, etc.

Ownership changes have an effect on the level of farmland fragmentation and also influence the economic efficiencies of farms. Economic efficiency in turn determines a farm’s competitiveness and position in the land market. Moreover, it is assumed that ownership change and competition are also significantly affected by the spatial differences in quality of farmland.

The evaluation of these assumptions is based on an explanatory model which is intended to help the understanding of system behaviour (cf. Bossel 2007). The model does not aim at an exact forecast, but strives to reveal key interrelations predominant in the system.

The aims pursued in this study are (i) to investigate interrelations of structural change processes in agriculture and conditions regarding location (fragmentation and quality of land) and (ii) to propose an object-oriented approach for SD simulations. In the next two sections the example of structural change in agriculture is briefly outlined and the object-oriented SD approach is conceptualised. Section 4 explains the use of this concept for modelling structural change. This is followed by a section on model data and simulation results in section 5. Finally, conclusions are presented and suggestions are made for possible future applications of object-oriented SD simulations.

## 2. Case study outline

The reasons for studying structural change are, among others, the anticipated negative effects of larger farms on species richness (Marini *et al*. 2009) and trends in rural demography and the tourism industry (Weiss 1998). A number of spatial models of the structural change of farms have been implemented (e.g. Freeman *et al*. 2009; Happe *et al*. 2006). These models focus on technological changes, public programmes and the socioeconomic and macroeconomic environment as key drivers of structural change documented in the literature (cf. Weiss 1998; Zimmermann *et al*. 2006).

As opposed to those implementations our model puts emphasis on the spatial implications of differences regarding location. The underlying assumption of the evaluation of the process-space interaction is that heterogeneity of the spatial configuration of farms has an influence on their resources. In other words, spatial characteristics of farms matter. Spatial heterogeneity among farms in our model is constituted by the fragmentation of land ownership and the distribution of farmland quality.

Fragmentation is an issue as it increases the required inputs in terms of transportation costs and working hours, whereas outputs stay the same. Large-scale farms may be able to overcome this problem by means of investments in high-performance machinery. Nevertheless fragmentation imposes limitations on farmland expansion and thus on structural change processes.

Effects of structural change on fragmentation are assumed to be dependent on initial conditions. An initially scattered pattern may evolve to a more consolidated pattern in terms of farm fragmentation. Moreover, a dynamic land rental market may have a consolidating effect on landholdings (Bizimana *et al*. 2004). Consolidated holdings, however, tend to become more fragmented as a result of scaling-up processes (cf. Dijk 2003; Edwards 1978).

Quality is a property of farmland that remains unchanged by the process of structural change. It does, however, influence the structural change process. Pronounced spatial differences in farmland quality may intensify structural change processes, whereas homogeneity gives rise to relative stability of the system. Moreover, Eitzinger (2007) assumed that increasing farm sizes may compensate for any reduction of agricultural yields. A substantial shift in local yield reliability may affect grassland farm sizes. We include hypothetical scenarios for the quality of farmland in the evaluations to show how changes in carrying capacities of farmland due to climate change can be taken into consideration.

The modelling of such a system requires the synthesis of process and spatial dynamics. This type of integration has previously been realised in different ways (e.g., Lauf *et al*. 2012; Walsh *et al*. 2013). A promising approach is the use of hybrid models, combining features of SD and agent-based models as conceptualised by Vincenot *et al*. (2011). The object-oriented approach for SD introduced in the following section follows a similar concept.

## 3. Object approach

The merits of raster and object view as representations of geographic space have been broadly discussed in the GIS community (e.g. Peuquet 1988; Couclelis 1992; Blaschke *et al*. 2000). The main conclusions of these discussions can be adopted to answer the question: why use an object view in SD simulations?

Firstly, the selection of an abstraction scheme is a matter of representation. Object models lend themselves nicely to the representation of discrete geographic features with well-defined boundaries. This practically applies for all human artefacts falling into two categories: (a) engineering works such as roads, bridges, dykes, railways or surveying marks, and (b) administrative and property boundaries (Couclelis 1992). Moreover, despite their fuzzy characteristics, natural features may be best represented as objects in cases where independent objects are meaningful in themselves and geometry is of importance to the question being investigated. For instance, the discretisation of geomorphic features is used as a method to classify landforms (e.g. Drăguţ & Eisank 2011).

Apart from the appropriate representation of geographic space, the alternative object view for SD simulations enables an application of computational operations and techniques inherent to this concept. Geographic features visually interpreted as objects are fully recognised as objects by the system. This allows the modeller to make use of intrinsic object attributes such as the length of a polyline or polygon perimeter and area (De Smith *et al*. 2007). In addition, relationships among objects may be stored as complementary topological information in a database. This concerns relationships between objects, used to define more complex objects (e.g. points forming a line), relationships between objects defined by their geometry (e.g. adjacency or connectedness) and other relationships between non-connected objects (e.g. distance) or directions of flow (Goodchild 1993).

In summary it can therefore be said that an integration of the object view in SD, on the one hand, allows for simulating the dynamics of spatially non-continuous phenomena. On the other hand, spatial relationships come in handy in cases where spatial geometries and relationships between discrete entities are an important input in the simulation.

**Figure 1.**
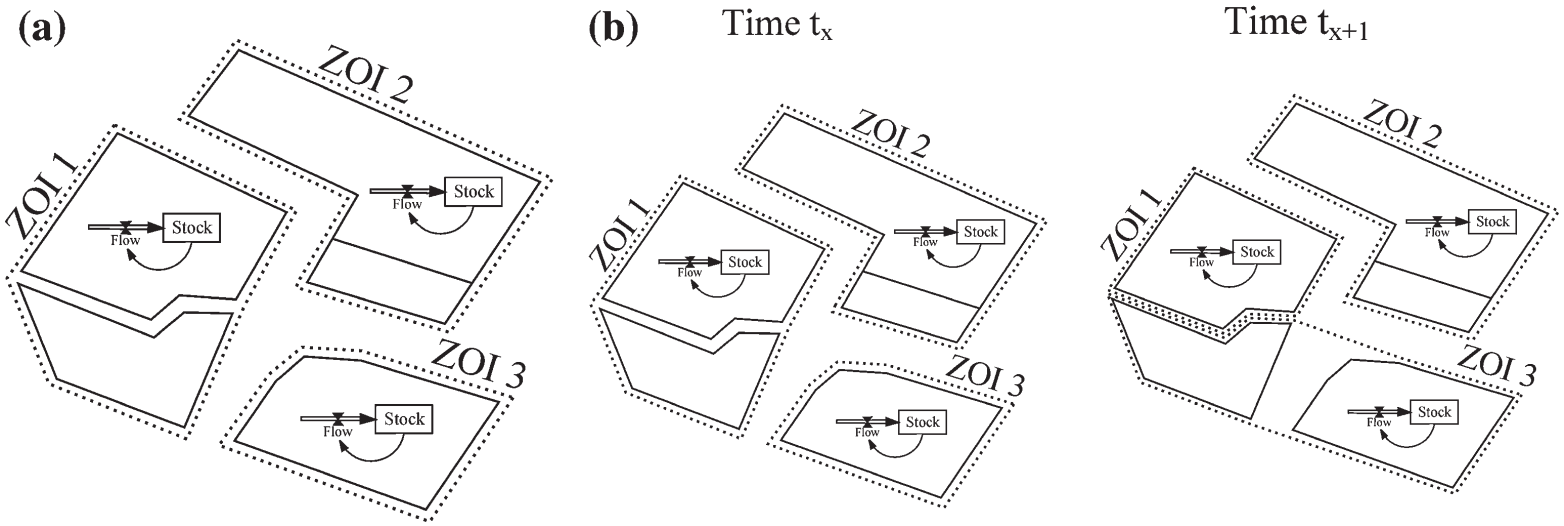
SD models are interfaced with spatial objects (zone of influence ZOI). (a) Object-to-model ratio is constant. (b) SD models communicate with a variable number of objects (variable zone of influence).

The interaction between processes and space inherent to such simulations may be expressed by linking SD process models to object models (see Figure 1). Spatially distributed processes are modelled by duplicating SD models which are linked to objects. The objects assigned to a process model define its zones of influence (ZOI) (cf. Vincenot *et al*. 2011).

Two types of interaction between SD and object model, i.e. between process and space, are distinguished: (i) the geometry and topology of objects affect processes modelled in SD; (ii) the geometry and topology of objects affect processes modelled and simultaneously processes modify object structures. In model type (ii) objects are exchanged between ZOIs as a process gains control over another. The latter case implies dynamic feedback between process and space.

An implementation of this model type makes high demands on the space-time association. The current state of spatial topology needs to be quantified by means of spatial analyses and updated in a spatial database. Therefore, operations such as process simulation (SD module), spatial analysis and data update (GIS module) are required to be tightly coupled and synchronised.

Recently a Python-based middleware program was presented to synchronise dynamic SD simulations with spatial analytical GIS operations (c.f. Neuwirth *et al*. 2015). The function of this tool was illustrated using the example of land cover change. Land cover patches (objects) were generated by assigning numeric identifiers to raster cells. One basic problem of this approach is that although objects can be visually interpreted, they are not recognised as objects by the computer system. Accordingly, the use of the aforementioned intrinsic object geometries and relations is very limited. Hence, while techniques for synchronising operations can be adopted, the raster view is replaced by an object view.

Moreover, whereas in the land cover model object size is directly bound to system stock variables, the presented approach uses an indirect link. This means the SD module doesn’t deliver object size as an output, but it simulates processes which may in consequence cause an expansion or contraction of the variable ZOI. In the case of a model of structural change in grassland farming, simulations of economic processes are run in SD. The economic success of a farm eventually determines the actions taken by the farmer on the land market. In other words, the SD simulation outcomes drive decisions which affect spatial patterns of ownership.

## 4. A model of structural change in grassland farming

According to the terminology used in section 3, farmland plots defined by the land-use cadastre are equivalent to objects in this example. Every farm comprises a certain number of plots. The aggregate of plots of a farm constitutes its ZOI. As farms compete for farmland, ZOIs are subject to modifications which in turn affect processes of structural change (e.g. due to changes in farming efficiency).

In order to model structural change as well as process-space interaction, a number of difference equations were defined and formalised in SD. Basic finance equations were modified from Balmann (1997) for calculating a farm’s economic situation over time (see Table 1).

**Table 1.** Farm model equations.

No.	Variables	Equations and descriptions	
(1)	Debts		
(2)	Repayment		is the repayment rate
(3)	Interest expenses	*IC* = *DL* ⋅ *i*	*i* is the interest rate
(4)	Assets	*A* = *I* − D	*I* are the investments
(5)	Depreciation	*D* = *A* − *i*_*D*_	*i*_*D*_ is the depreciation rate
(6)	Rent income		*Area*_*l*_ is the area of a plot leased *r* is the rent per unit of area
(7)	Rent expenses		*Area*_*r*_ is the area of a plot in rent
(8)	Transportation costs		*DT* is the distance travelledare the costs per distance travelled
(9)	Number of animals		*Area* is the area of a plot being cultivated*CC*_*P*_ is the carrying capacity of a plot expressed as the maximum number of animals per unit of area
(10)	Turnover	*T* = *N* ⋅ *P*	*P* is the price gained per animal and time step
(11)	Profit	*Y* = *T* − *D* − *IC* − *RC* + *RY* − *TC*	
(13)	Equity capital		
(12)	Withdrawals		*i*_*WD*_ is the withdrawal rate
(14)	Liquidity		

^*^ The rent is negotiated individually for every plot in an auction.

^**^ The distance travelled per time step is estimated based on network distances.

^***^ Carrying capacity serves as a measure of farmland quality.

This system of non-spatial difference equations was supplemented with travel distances derived from a network (equivalent to model type (a) in Figure 1). Distances between farmstead and plot were calculated for every landholding. The distance travelled (DT) is calculated as the sum of individual farmstead-plot distances (see Table 1; equation 8) and serves as an estimate of actual travel distances.

In addition, a set of rules has been determined. For instance, farm operators can raise credits as a 1:1 share of equity to invest in assets. Investments are linearly related to the farm size as the stock of machinery and buildings is assumed to be constant per unit of area (cf. Freeman *et al*. 2009). Investment assets are continuously depreciated over five years in the simulation.

Moreover, rules on the auctioning of farmland have been defined. Farms auction all of their plots in the case of bankruptcy (Liquidity <= 0). Therefore, auctions only take place if farms became bankrupt. In addition, auctioning of farmland due to the absence of a farm successor was implemented as an optional model setting. Ageing of farmers is implemented by simply counting time steps in the simulation. Once a farmer turns 65, farm succession is evaluated as a derivative of the farm’s carrying capacity *CC*_*F*_ based on the following logistic regression adapted from Stiglbauer and Weiss (1998):(15) 
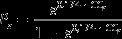



Regardless of what causes the compulsory auction (bankruptcy or the lack of a farm successor), farmland is not sold, but leased to other farmers. Once a plot is leased, it remains with the renter unless the renter becomes bankrupt or cannot find a successor himself. In this case leased plots as well as owned plots are put up to auction.

The potential of a farmer to rent farmland in addition crucially depends on workforce capacities. It is assumed that farms are small-scale and family-operated, which implies that the farm-owned labour is about the same for every holding. Nevertheless, reinvestment of profits in machinery with higher performance diminishes operational efforts. This concerns the adoption of technologies suitable for operating the respective farmland. As a consequence, the potential (*Pot*) of a holding (*i*) to rent land in addition may be calculated as a normalised function of profits (*Y*) and operational efforts (*E*):(16) 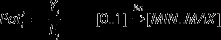



Operational efforts are assumed to be highly dependent on different aspects of land fragmentation such as plot shape, plot size, distances and spatial distribution of plots. In order to take this effect into consideration, a dimensionless fragmentation index was derived for every farm (see section 'Quantification of fragmentation'). Efforts (*E*) were computed as the product of this index and farm size.

High profits associated with low operational efforts indicate high potential for investments on the land market. High potentials increase the willingness of farm operators to pay rents above market prices. The theoretical maximum bid a farmer can offer in an auction (*r*_*max*_) is defined as the difference between expected additional gross margin and farm-specific transportation costs (Balmann 1997). This can be calculated according to the formula(17) 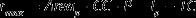



where plot area (*Area*_*p*_) and required investment assets for a plot (*I*_*p*_) are introduced as new variables. The maximum rent is now scaled down by individual farm potentials as follows:(18) 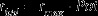



whereby *r*_*bid*_ is a farmer’s bid for renting a plot. If nobody can bid more than zero, the plot is rented at no cost to the farmer with the lowest negative bid. In addition, an optional model setting is implemented which allows the abandonment of such unprofitable farmland.

The way bids are calculated ensures that distance to plots limits their profitability. It also implies that farms benefit from bankruptcies in their vicinity as this enables farmland expansion at low cost and minor fragmentation. However, farms with higher potentials may outbid farms which are closer to plots for auction.

At the same time farm potentials may decrease as a function of farmland rented, as expansion is often associated with increasing farmland fragmentation and operational efforts. In order to model effects of size and fragmentation, spatial attributes need to be updated in the simulation whenever plots are reassigned (equivalent to model type (b) in Figure 1). This concerns in particular the calculation of transportation costs as well as farmland fragmentation.

### Quantification of fragmentation

Fragmentation in agriculture is related to operational efforts, as outlined in the previous section. The question to be answered is what makes cultivation of farmland more time-consuming and inefficient. Edwards (1978) stressed the importance of headquarter-plot distances and the number of plots. Furthermore, the dispersion of plots in space as well as their size and shape characteristics contribute to fragmentation (King & Burton 1982).

A highly fragmented farm is composed of many small plots which are dispersed in space, oddly shaped and far away from the farmstead. By contrast, consolidated farms exhibit large rectangular plots which are clustered in space and close to the farmstead. Hence, fragmentation is defined and evaluated in this study by taking the following fragmentation variables into account: (i) shape of plots, (ii) size of plots, (iii) between-plot dispersion and (iv) farm-to-plot dispersion.

The shape of plots was evaluated based on a multi-parameter index proposed by Demetriou *et al*. (2013) and supplemented with an additional plot size parameter introduced in Demetriou *et al*. (2012a). They derived multiple parameters which were defined by five land consolidation experts based on the direct value rating method (see also Demetriou *et al*. 2012b for more details on the value rating method). The parameter values are dimensionless scores between 0 and 1. A value of 1 indicates an ideal rectangular plot whereas a low value indicates the opposite.

**Figure 2.**
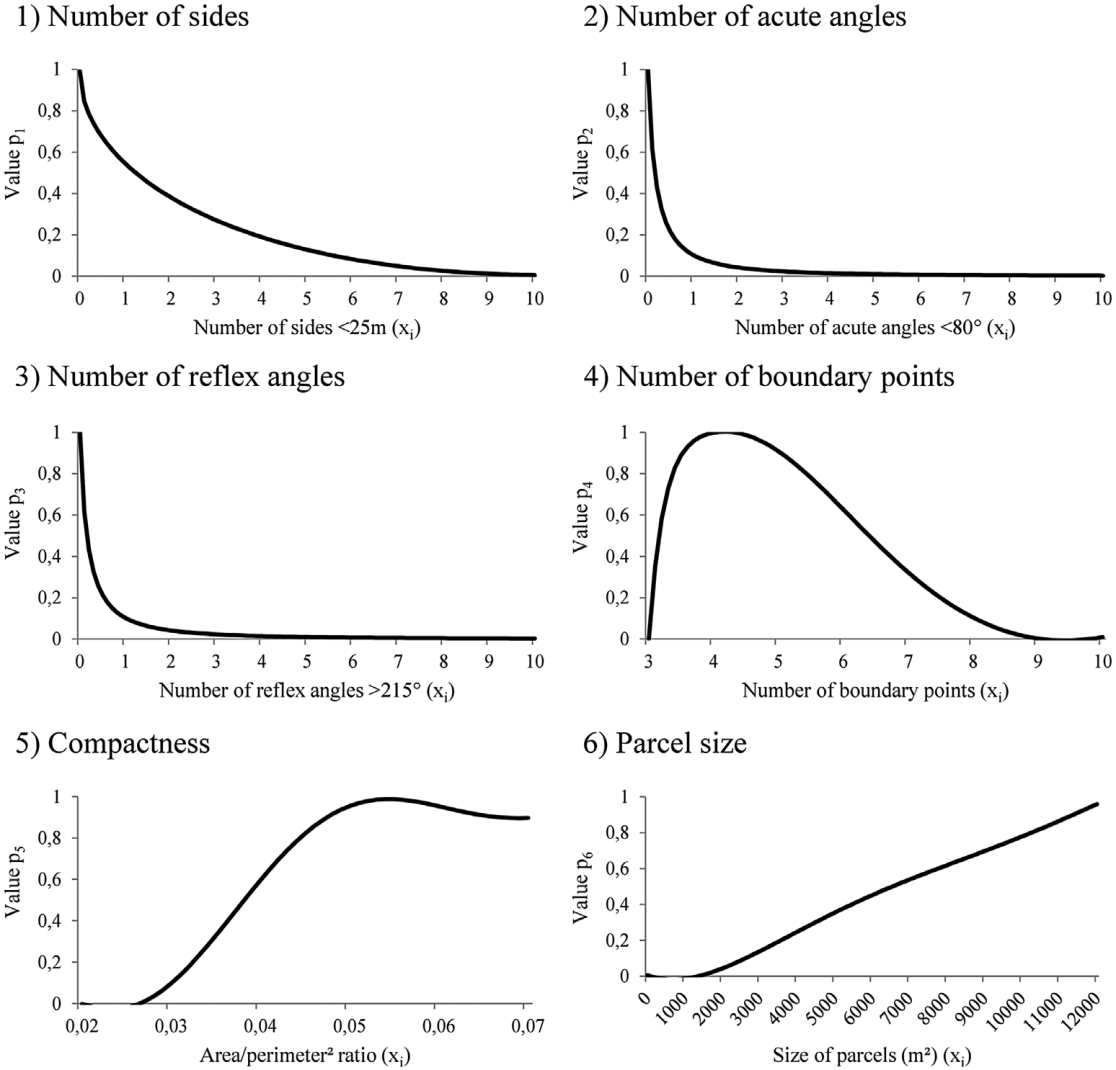
Parameter value functions (cf. Demetriou *et al*. 2012a, 2013).

The index comprises six different parameters which are defined by (1) the number of sides with a length less than 25 m, (2), the number of acute angles of less than 80°, (3), the number of reflex angles greater than 215°, (4), the number of boundary points (corners of a plot), (5) compactness defined as plot area to squared plot perimeter ratio and ( 6) plot size. Each of those parameters is standardised by means of an individual value function (see Figure 2).

The index is calculated for every plot by summing up standardised parameters (*p*_*i*_) and dividing them by the number of parameters involved. In order to put emphasis on certain characteristics single parameters may be complemented with additional weightings (*w*_*i*_). The parcel shape index is obtained as follows:(19) 
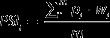



In addition between-plot distances were calculated using Dijkstra’s shortest network path algorithm (cf. Dijkstra 1959). The between-plot dispersion of a farm is computed by(20) 
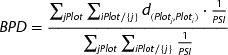



where *d* is the shortest network distance between plot centroids and *PSI* is the plot shape index of the destination plot. Similarly, farm-to-plot dispersion is calculated for every farm as follows:(21) 
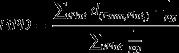



In this way highest *BPD* and *FtPD* indices were calculated in cases where plots of a farm are far apart from each other or distant from farmsteads, small scale and complexly shaped. Between-plot and farm-to-plot dispersion are summarised and normalised by scaling between 0 and 1 in the model to get comparable measures of single farm fragmentation (see equation 22).(22) 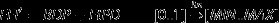



Similar between-plot dispersion, farm-to-plot dispersion or plot weighting metrics found application in numerous fragmentation analyses. A complete list of metrics which have inspired the definition of equations 20, 21 and 22 is presented in conjunction with other examples in the appendix.

The update of this fragmentation index and operational efforts is performed for every farm renting plots in addition after an automatic temporal interruption of the SD simulation. This enables an integration of immediate feedback between economic and spatial structural processes.

### Input data

An area north of the Austrian Alps which encompasses five cadastral communities (Hof, Kirchberg, Neuhofen, Plainfeld, Schwaighofen) and an area of about 30 km² was chosen as a study site to investigate anticipated process-space relations. The predominance of grassland agriculture allows the exclusion of agricultural land use changes in the model and to focus on the evolution of property relations as one aspect of structural change. Moreover, the hilly terrain implies complex, non-uniform property relations which make fragmentation a relevant aspect of economic efficiency in this region.

The investigation of anticipated effects (see section 2) requires simulations performed on a farm level. Unfortunately, the use of data at this level is restricted due to privacy regulations or fine-grain data are missing. In order to overcome this problem, pseudo-data sets were generated from basic, real-world data.

Cadastral polygon data including 414 agricultural plots as well as 24 farmsteads were provided by the Austrian Federal Office of Metrology and Surveying as an anonymised data set without information on property relations. Furthermore, statistical farm size distributions were acquired freely on a national level. This statistic could be used to supplement cadastral data with missing information on ownership relations.

The procedure used to recreate ownership relations was based on a simple seed-growth algorithm which was implemented in Python. Firstly, statistical farm size values are randomly assigned to farmstead polygons (seed) to set desired farm sizes for every farm. Subsequently, plots closest to the respective farmsteads are assigned consecutively until the desired farm size criterion is met. In this way, the statistical farm size distribution can be approximated (see Table 2).

**Table 2.** Comparison of statistical and pseudo size distributions.

Farm type	Farms per type [%]		
	Stat. 1999^a^	Level 1^b^	Shuffled 50^c^
< 10 ha	33.21	46.41	44.63
10 < 20 ha	21.37	21.42	23.21
20 < 50 ha	31.14	23.2	21.42
>50 ha	14.26	8.91	10.7
Goodness of fit (χ²)		2.22 (*p* > .05)	1.92 (*p* > .05)

^a^ Statistics Austria (2013).

^b^ Closest plot assignment.

^c^ 50 plots shuffled.

Alternatively, the fragmentation of landholdings can be increased by assigning second, third or fourth closest plots to farmsteads. This *selection parameter* was foreseen as a user input field in the graphical user interface of the *ownership data generator*. Furthermore, fragmentation may be increased considerably by shuffling plots. This was implemented by a random selection and reassignment of a user-defined number of plots.

The output of the *ownership data generator* was fed into a GIS database, which was later interfaced with the simulation modules. The simple spreadsheet-like database comprises fields holding information on plot owners and tenants as well as plot rents. Initially, every plot is owned and farmed by the same farmer. Therefore, fields giving information on the rental situation were kept empty. In addition to initial ownership and rental conditions, fields such as plot area and quality were inserted. Plot sizes are calculated automatically as a built-in function of the database. The quality database field and values had to be supplemented manually.

**Figure 3.**
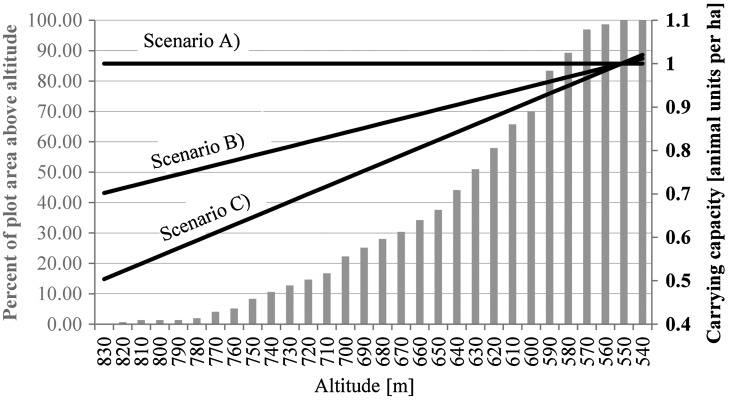
Hypsometric distribution of plots and height-dependent farmland quality scenarios A, B and C.

Quality of farmland plots was modelled as a function of average altitude using three hypothetical scenarios (see Figure 3). Grassland quality is expressed in animal units per hectare of land (au ha^−1^), which serves as a measure of grassland carrying capacity. An animal unit is equivalent to a 454 kg animal, which roughly corresponds to a mature cow (Scarnecchia 1985).

The three scenarios are hypothetical and based on simplified assumptions.

In Austria the average stocking density corresponds to about 0.8 au ha^−1^ (Buchgraber & Schaumberger 2006), which is assumed to be roughly in line with the physical limitations of natural carrying capacities. This statistic, however, also includes extensive upland areas which exhibit significantly lower carrying capacities. Hence, base scenario A assumes a slightly increased and uniform carrying capacity of 1 au ha^−1^. In order to account for regional variation, values can be lowered with altitude. Scenarios B and C hypothesise a linear reduction of carrying capacity to 70% and 50% at highest altitudes, respectively.

The lowest carrying capacities can certainly be expected for the highest plots. Nevertheless, climate change may have a positive effect on the length of growing seasons and productivity of upland areas (Schaumberger 2011). Hence scenario A may be interpreted as a climate change scenario which assumes that climate change balances out the diversity of growing seasons. Scenario B depicts moderate climate change impact, while impacts in scenario C are negligible.

Further model parameters such as the initial age of farm operators could be derived from census data (c.f. Statistics Austria 2013). The statistical age distribution of Austrian farmers from 1999 is used to approximate the age of the 24 farm operators in the study region by percentage.

Moreover, transportation costs are assumed to have considerable influence on spatial patterns produced by simulations. Hence, multiple scenarios with different settings are run to allow for an evaluation of parameter impact. Due to the high variability of cost figures and general lack of reliable data, expert consultations were undertaken to set parameters as plausibly as possible. Other exogenous input parameters are also based on expert opinions as data are not available on a small scale or inputs are purely behavioural in nature and thus empirical data are rarely available (see Table 3).

**Table 3.** Exogenous parameters, values and units.

No.	Parameter	Value	Unit
(1)	Initial debts; *DL*_*t*=*0*_	0.0	cu^*^
(2)	Repayment rate; *i*_*r*_	0.1	-
(3)	Interest rate; *i*	0.05	-
(4)	Asset investment	700	cu ha^−1^
(5)	Carrying capacity of a plot; *CC*_*P*_	See Figure 3	au^**^ ha^−1^
(6)	Prices; *P*	200	cu au^−1^ y^−1^
(7)	Withdrawal rate; *i*_*WD*_	0.7	-
(8)	Credit to equity share	1:1	-
(9)	Initial age of farmers	Census data	y
(10)	Transportation costs; *DC*	Scenario-dependent	cu km^−1^

^*^ currency unit.

^**^ animal unit.

Parameter estimates are suitable for analysing general dynamic patterns produced by this complex system. The correct identification of important causal links is more relevant to understand the system’s dynamics than a time-intensive empirical validation of dependencies, which would go way beyond the scope of this study (c.f. Bossel 2007). The model is designed to run simulations for deriving tendencies as a rule of thumb.

## 5. Results

### Fragmentation of farmland and structural change

**Figure 4.**
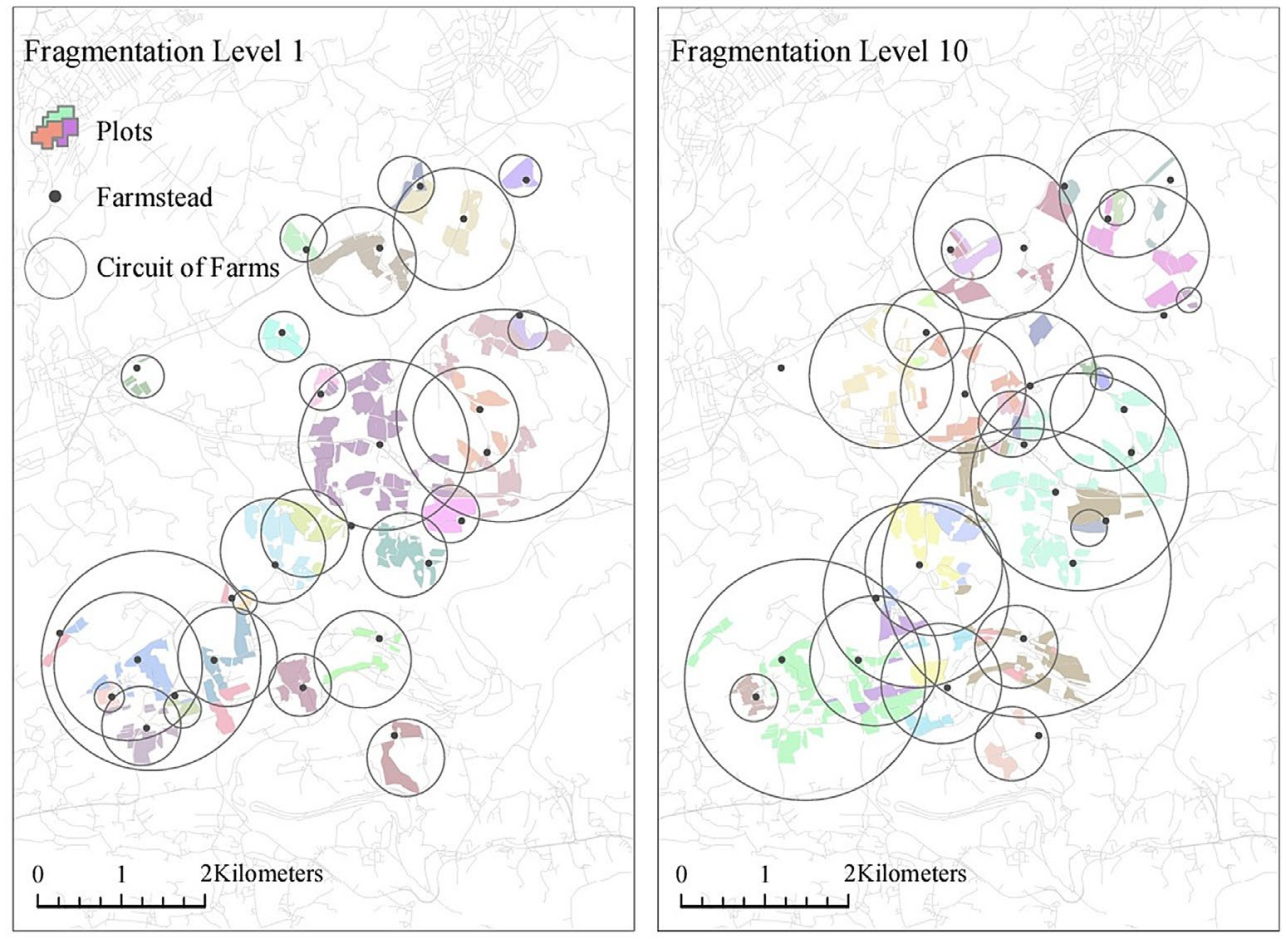
Input fragmentation data created by means of the ownership data generator; left: minor fragmentation (selection parameter value 1); right: pronounced fragmentation (selection parameter value 10).

Structural change was modelled for 15 years based on 10 synthetic datasets created by means of the ownership data generator (see Figure 4). Selection parameter values between 1 and 10 (fragmentation levels 1 to 10) were used to produce distinct initial conditions in terms of fragmentation for each simulation. The carrying capacity of grassland plots corresponds to scenario A (see Figure 3). Moreover, high (10 cu) and low transportation cost (5 cu) simulations were compared. Thus, 20 simulation runs were conducted to reveal effects of structural change processes on fragmentation under varying initial conditions.

**Figure 5.**
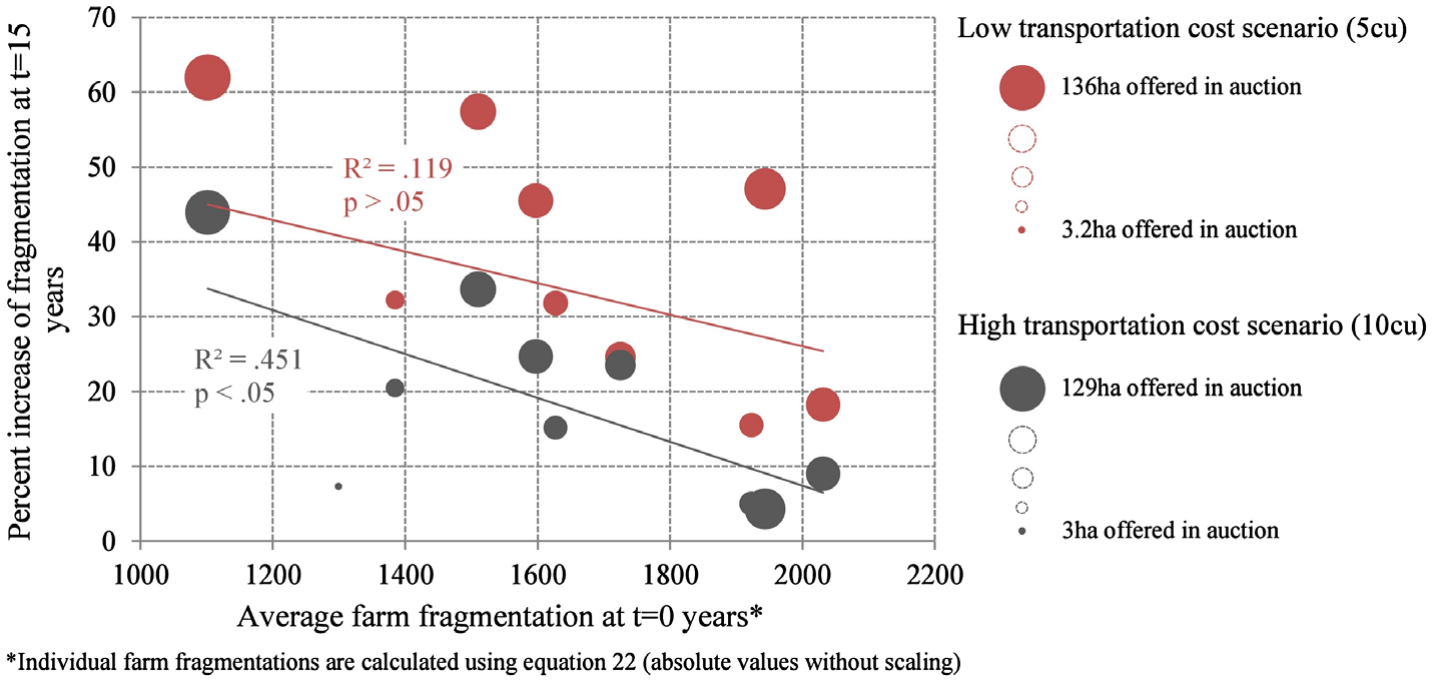
Effects of initial fragmentation and transportation costs on the evolution of fragmentation due to structural change.

The simulation results confirm the assumption of increasing fragmentation due to structural change. However, the rise in average farm fragmentation is affected by initial fragmentation and transportation costs. A significant correlation (*p* < .05) exists between initial fragmentation and increase in fragmentation under high transportation cost conditions (10 cu). Simulations initialised with consolidated fragmentation patterns tend to show higher fragmentation gains than those initialised with highly fragmented structures. This dependency was not significant under low transportation cost conditions (5 cu) (see Figure 5).

Transportation costs limit fragmentation induced by structural change processes. Plots provided in auctions are rented to holders in the closer vicinity as transportation costs become economically relevant in the high transportation cost scenario (10 cu). In this way transportation cost determines future pathways of fragmentation.

In contrast, the amount of land provided for rent in a simulation period proved to have negligible effects on the evolution of fragmentation. Auctioned land is also independent of initial fragmentation conditions or transportation costs. Variations in the amount of land offered in the respective simulation runs are caused by the probabilistic nature of the farm successor logic (see equation 15 in section 4).

Alternatively, initial fragmentation conditions were significantly increased in order to identify the fragmentation level above which structural change causes farmland consolidation. Highly fragmented property relations were generated by using the shuffling function of the ownership data generator. Whereas selection parameter values were kept constant at 1 (level 1), the number of plots being shuffled was varied between 10 and 100 with an interval of 10. In this way 10 different datasets were generated. The shuffling significantly increases the fragmentation of landholdings, since proximity is ignored in the reassignment of plots. Simulations conducted on these data sets were run for 15 years assuming high transportation cost conditions.

**Figure 6.**
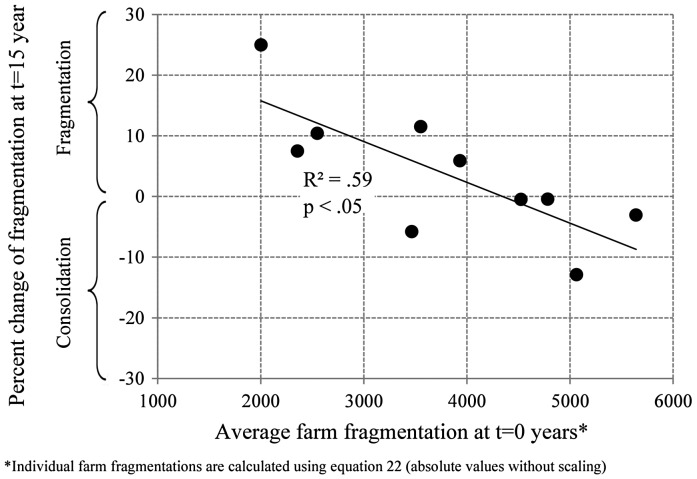
Effects of initial fragmentation on the evolution of fragmentation due to structural change under highly fragmented initial conditions and high transportation costs (10 cu).

Analogous to previous simulations, the highest fragmentation increase rates were observed for simulation runs initialised on lower fragmentation levels. Simulation runs conducted based on highest average fragmentation levels, however, show a consolidating effect of structural change processes (see Figure 6).

**Figure 7.**
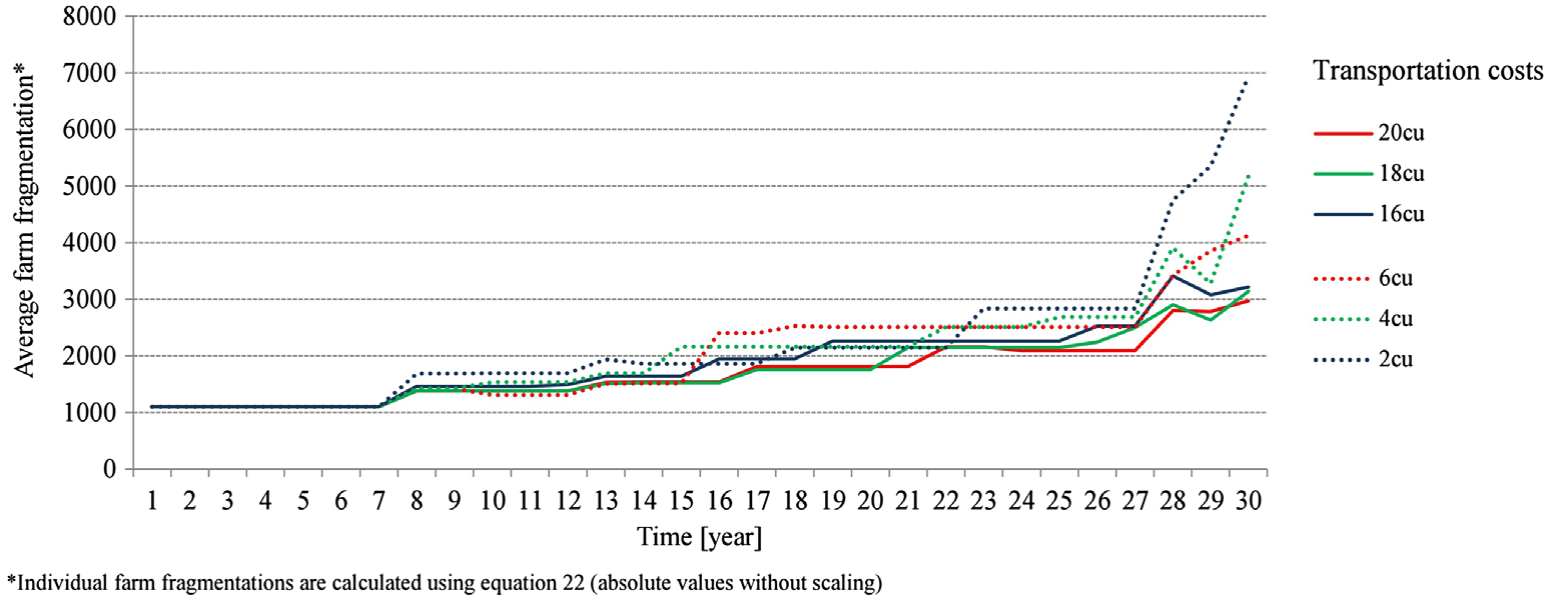
Change of average farm fragmentation over time under different transportation cost conditions; simulations are based on fragmentation level 1.

In order to examine the effects of transportation costs over time, six additional simulation runs were conducted using fragmentation level 1. The range of transportation cost values was significantly increased (4 to 20 cu) to illustrate its effects on fragmentation patterns. The results show that the pace of change accelerates in the long run. While after 15 years average fragmentation was not even doubled in any of the scenarios, fragmentation increased 5-fold and above after 30 years (see Figure 7). This acceleration of fragmentation increase is much more pronounced in scenarios initialised with lower transportation costs (2 to 6 cu).

The time lag can be explained by effects of scale. At the beginning of the simulation bankruptcy assets are distributed among many small farms, whereas at the end of the simulation most of the land put up for auction is rented by a few large-scale farms. This inevitably leads to higher fragmentation increments over time. Low transportation costs exacerbate the situation, as plots of farmland remain profitable for large-scale farms even if they are distant from farmsteads. This mechanism could easily lead to an underestimation of long-term effects of varying transportation costs on the increase of farm fragmentation.

It may be concluded that structural change processes affect the evolution of farm fragmentation as a function of initial fragmentation and transportation costs. Structural change tends to just slightly increase fragmentation of highly fragmented farms or even decreases their fragmentation. Consolidated farms, however, become more fragmented. Thus, results are consistent with the original assumptions formulated in section 2.

Feedback between the state of fragmentation and future changes in fragmentation may be explained by the constraints of compact development. The capability for further compact development continuously decreases with compactness, whereas fragmented patterns give room for compact development. This implies that outwards development prevails for compact farms while fragmented farms may become more compact.

Contrary to our expectations, fragmentation is independent of the amount of farmland offered in auctions. A highly dynamic land rental market alone doesn’t foster land consolidation. Rather, a dynamic land market in combination with high transportation costs is responsible for a pronounced consolidation effect.

High transportation costs imply a steep increase of marginal costs with distance. As minimising travel distances becomes a relevant factor in economic success, farmers tend to rent farmland closer to their farmsteads. Therefore, fragmented landholdings tend to become consolidated under high transportation cost conditions.

### Quality of land and structural change

Quality scenarios A, B and C (see Figure 3) were run for 30 years to reveal the effects of quality patterns on structural change processes. Fragmentation level 1 was chosen as the input data set. High transportation costs (10 cu) were kept constant to foster consolidation. In addition to previous simulations, abandonment of uneconomic plots was considered as an alternative opportunity of action in the following simulation examples. Furthermore, the ageing of farm operators was turned off to avoid auctions as a result of overageing. The aim of the simulations was to reflect the economic consequences of changing quality patterns.

**Figure 8.**
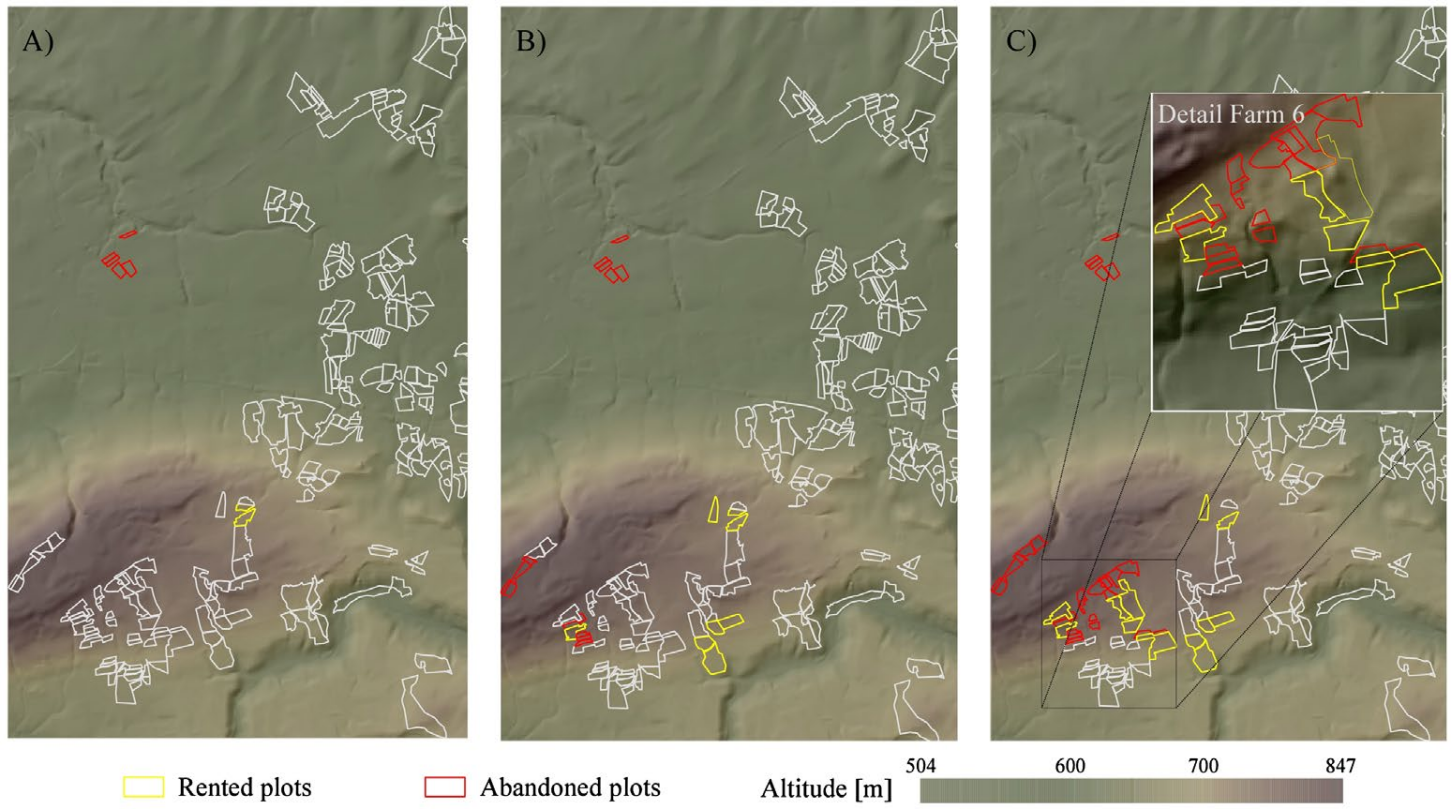
Farmland abandonment and renting situation at simulation time step *t* = 30 for quality scenarios A, B and C.

The number of bankruptcies increased with the increase of the quality function (see Figure 8). In other words, the number of bankrupt farms correlates to the advance of unfavourable conditions for upland farms. Apart from one farm in the northwest of the study area, bankruptcies were restricted to farms owning significant amounts of land in upland regions. Thus, quality patterns crucially affected structural change processes in the simulation. The highest dynamics were observed where quality of farmland was lowest.

Farms which were located next to these hot spots benefited from a large supply of affordable land. For instance farm 6 managed to rent the most profitable upland plots, which improved the economic situation of this farm in the long run (see Figure 8, detail view). This effect was reinforced due to a lack of strong competitors in the vicinity of farm 6, which enabled the renting of high-profit plots at low costs.

In contrast, unfavourable plots were abandoned as their profit margins were too low. The simulations showed that especially small plots which are distant from farmsteads were abandoned. Therefore, the relation between farm-to-plot distance and plot size appeared to be the most relevant factor. This is also underlined by the abandonment of a farm located in the north-western lowlands in all scenarios. Despite the high quality of farmland, renting doesn’t pay off for any holding as plots are too small and remote. Farmland abandonment also has a consolidating effect on holdings and thus fosters overall economic efficiency in the simulation.

As expected, pronounced spatial differences in farmland quality intensify structural change processes. Since the carrying capacity of grassland typically correlates to altitude on a regional scale, ownership dynamics occur especially along altitudinal belts. In contrast, homogeneity gives rise to relative stability in the system.

The assumed compensational capabilities of farms only hold true for farms on a medium altitude. Upland farms could not compensate for unfavourable conditions by renting profitable farmland in addition, as farms owning those high-profit plots were expanding themselves. Farms on a medium altitude, however, even increase their profits as compared to high farmland quality scenarios. This is due to the farmer’s cherry-picking behaviour revealed by the simulation.

These findings may also be relevant in the context of climate change. Climate change is on the whole likely to reduce grassland productivity in areas which are already susceptible to drier summer seasons and thus increases pressure on grassland farms to become more efficient (Neuwirth & Hofer 2013). Therefore, the lease or abandonment of single plots which are distant from the farmstead may increasingly become relevant in the future as a means to improve efficiency. Climate change may simultaneously improve the grassland productivity of upland areas in Alpine regions (Schaumberger 2011), which would at the same time mitigate the previously described topographic effects on competition.

## 6. Prospects of the object-oriented SD approach

Using object-oriented SD is essentially a matter of the appropriate representation of geographic space. For instance, an object-oriented SD model for the assessment of urban residential development was presented by Xu (2011). The number of houses demanded by a growing population was modelled in SD. Subsequently, required real estates could be allocated as 3D objects on a GIS map, which facilitates the interpretation of the predicted city design by spatial planners and decision-makers.

Moreover, the use of an object view facilitates modelling of feedback and co-evolution of process and space. To investigate the relation between space and process, it is often helpful to describe space in quantifiable terms (Hargis *et al*. 1997). The quantification of space is essential to establish criteria for relating spatial structures to their consequences (Levin 1992). The object-oriented spatial view enables an efficient analysis of spatial metrics and thus opens up a wide field of new applications of the SD concept in cases where contextual and spatial information is needed.

For instance, the allocation of real estates in the city growth model implemented by Xu (2011) could be made dependent on existing structures. City development is related to infrastructure networks and accessibility. Therefore, allocation of real estates may be modelled as a function of travel distance and time. The evolution of residential areas may in turn result in the development of new traffic infrastructure. This implies that distance metrics need to be re-evaluated over and over again to keep track of a city’s traffic infrastructure development.

The object-oriented SD approach enables the implementation of sophisticated origin-destination network analysis in such a simulation. Thus, object orientation may especially be of use in the fields of transportation research or spatial planning. Furthermore, phenomena related to human mobility such as the spread of infective disease (e.g. BenDor & Kaza 2012), CO_2_ emissions, resource consumption or social factors such as the quality of life could be modelled.

Moreover, object orientation may also be of relevance in the field of ecological modelling. The interrelation of process and spatial patterns has been one key focus of ecological research (Turner 1989). For instance, a number of landscape metrics were developed in a GIS environment to investigate the relation between landscape structure and biodiversity (Schindler *et al*. 2008). Also, many articles in landscape ecology use landscape metrics for habitat analyses (Uuemaa *et al*. 2009). The presented approach enables an integration of those metrics in dynamic SD-based simulations.

Similarly, spatial landscape metrics are used as an additional input parameter for simulating land use / cover change (e.g. Herold *et al*. 2005; Zamyatin & Cabral 2011). The use of SD for modelling land use / land cover change was demonstrated by Lauf *et al*. (2012). The integration of object metrics may contribute an important new level of information to SD-based land use / cover change modelling.

Object-oriented SD may also be of interest in socio-ecological systems science. A possible line of research could be the investigation of anthropogenic fragmentation of landscape, expansion of infrastructure, ecosystem degradation and resilience (e.g. Forbes *et al*. 2009). Likewise, human impact, ecological consequences and effects on human activity patterns could be treated as a spatial feedback system.

In summary, the approach benefits the modelling of systems involving dynamics in cadastral tessellations or any kind of mappable features and irregular spatial arrangements such as urban infrastructures, administration units or any anthropogenic landscape disturbances. The presented approach shall promote the use of SD concepts for those applications.

## 7. Conclusion

This study combines SD simulation with GIS-based object representations and spatial indices for modelling the effects of farmland fragmentation and quality in relation to farm structural change. The simulations reveal a control loop inherent in the evolution of farmland fragmentation over time. Simulations conducted under high initial farmland fragmentation conditions resulted in minor increase of fragmentation or consolidation, while consolidated farmland tends to become highly fragmented.

This is explained by the spatial limitation of compact development. A compact farm may be restricted to radial development, which increases fragmentation and decreases economic efficiency. A fragmented farm, however, may have the chance to consolidate its farmland by renting additional plots close to the farmstead. This is additionally promoted by increasing costs per distance travelled (e.g. increase in fuel prices). Therefore, structural change may turn fragmentation into consolidation provided enough land is offered on the land market, transportation costs are high and the spatial configuration of ownership (fragmentation) allows for compact development. In addition to consolidation achieved by renting plots, simulations also suggest favourable effects associated with the abandonment of marginal or unproductive plots.

Furthermore, differences in grassland production reinforce structural change. Pronounced differences in quality between upland and lowland plots lead to the bankruptcy of upland farms, which fosters a highly dynamic land market. Farms located at a medium altitude which are close to these hot spots benefited from a large supply of affordable land. This effect is especially relevant for agricultural regions characterised by complex terrain. Under such preconditions bankruptcy assets are often only relevant for a few bidders, which leads to cherry-picking behaviour and abandonment of unfavourable plots.

The simulation demonstrates the successful integration of GIS-based object representations and spatial analysis in an SD modelling framework. The approach makes use of intrinsic object geometries (e.g. plot shape) and relations between objects (e.g. farm-to-plot network distance). Attributes of dynamic space are updated in the simulation, which ensures immediate process-space feedback in the model.

In this way established concepts of SD modelling are adopted for conducting object-oriented discrete event simulations. The same approach could potentially find application wherever object geometries or object relations are mutually interrelated with continuous processes (e.g. ecological modelling). The object view also lends itself nicely in cases where space is better represented by discrete entities (e.g. transportation and infrastructure planning).

Nevertheless, alternative applications would require amendments to the approach. The presented dynamic modelling of farm sizes could be extended by including line objects to represent dynamics in network space. Moreover, in its current form linking multiple SD process models to a single object is not foreseen. This would also raise questions on the integration of process and object hierarchy which were not considered in the current version.

Furthermore, the object approach produces a large computational overhead and thus aggravates sophisticated sensitivity testing by means of Monte Carlo simulations. This overhead is not a result of context switching between GIS and SD applications, but a consequence of the iterative use of spatial analytical GIS operations. In the presented simulations constraints on computational efficiency are especially an effect of costly network analysis performed on high numbers of origin-destination pairs. The alternative use of specialised routing packages such as, for instance, Python Networkx may significantly improve performance. For a more detailed discussion of this issue the performance analysis presented in Neuwirth et al. (2015) can be consulted.

Apart from issues pertaining to the technical implementation, the presented concept constitutes a natural way of modelling phenomena whose behaviour is affected by spatial structure or topology. Object shape, size, orientation and movement in networks are best represented by making use of intrinsic geometry attributes and object relations in vector data. Process models can be linked to these realistic object representations of physical structure, which at the same time constitute a ‘template’ for the SSD model’s modular assembly.

A possible avenue for future research may be the additional embedding of individual-based modelling (IBM) capabilities. The object-oriented SD approach is not entirely equation-based. A set of rules on farmland auctioning and pricing was defined in the example presented. Combining equation-based SD with rule-based IBM is expected to represent dynamic phenomena in a more natural and efficient way.

## Funding

This work was supported by Austrian Science Fund (FWF) [grant number DK W 1237-N23].

## Appendix

Table A1. List of other fragmentation indices being reviewed for this article.

**Table:**

No.	Indices	Calculation	Considered aspects of fragmentation	Reference
(1)	Average farm size		Relative proportion of the holding	Thompson (1963)
(2)	Mean number of plots		Number of parcels, parcel size	Bizimana *et al*. (2004)
(3)	Simmons’s Index		Farm size, number of parcels and parcel size	Simmons (1964)
(4)	Simpson’s Index		Farm size, number of parcels and parcel size	Simpson (1949)
(5)	Schmook’s Index		Farm size and spatial distribution of fields	Schmook (1976)
(6)	Igbozurike’s Index		Number of parcels, parcel size, farm-plot distance and between-plot distance	Igbozurike (1974)
(7)	Weighted mean center of parcels		Number of parcels, spatial and value distribution of fields	Demetriou *et al*. (2012a)
(8)	Weighted dispersion of parcels		Number of parcels, spatial and value distribution of fields	Demetriou *et al*. (2012a)
(9)	Gross margin per holding		Parcel shape and size, number of parcels, distinction between working costs and travel costs, spatial distribution of fields, farm-plot distances	Gonzalez *et al*. (2007)

Farm size *A*; plot size *a*; average plot size ; number of farms *N*; number of plots belonging to a holding *n*; farm or plot index *i*; area of convex hull *A*_*CH*_; distance covered by the operator in a single round that takes him to all of his parcels *D*_*R*_; coordinates of the weighted mean center  and ; coordinates of the parcel centroids *x* and *y*; parcel weight (e.g. value of field) *w*; weighted dispersion of parcels *WdoP*; gross margin *GM*; useful area *Ua*; expected yield *y*; price *p*; tillage time required *Tt*; cost per time of operation *C*_*T*_; transportation distance *Td*; cost per travel distance *C*_*T*_.
